# Feeding and Growth in the Ephyra Stage of *Aurelia coerulea*: An In Situ Study

**DOI:** 10.3390/biology14060687

**Published:** 2025-06-12

**Authors:** Seo Yeol Choi, Kyoung Yeon Kim, Seok Hyun Youn

**Affiliations:** Oceanic Climate and Ecology Research Division, National Institute of Fisheries Science, Busan 46083, Republic of Korea; seoyeol84@gmail.com (S.Y.C.); weedy7411@korea.kr (K.Y.K.)

**Keywords:** jellyfish, ephyra stage, field experiment, zooplankton grazing, population recruitment, temperate coastal ecosystem

## Abstract

The ecological role and in situ feeding potential of *Aurelia coerulea* ephyrae remain poorly quantified. We conducted in situ chamber experiments in Geoje and Jaran Bay, Korea, to evaluate clearance rates, specific growth rate (SGR), and grazing carbon among ephyrae of different sizes and origins. Larger field-collected ephyrae had higher clearance rates (4.2 ± 0.8 mL ind^−1^ h^−1^) and grazing carbon (17.2 ± 1.0 μg C ind^−1^ d^−1^) than smaller individuals (1.6 ± 0.5 mL ind^−1^ h^−1^; 5.2 ± 1.1 μg C ind^−1^ d^−1^), though the differences were not statistically significant (*p* > 0.05). Field-collected ephyrae exhibited significantly greater clearance (*p* < 0.05) and grazing carbon (*p* < 0.01) than laboratory-reared ephyrae, while laboratory-reared ephyrae had higher SGR (*p* < 0.01).Grazing efficiency did not differ significantly between groups (*p* > 0.05). SGR peaked at intermediate prey concentrations (0.189 ± 0.013 d^−1^, *p* < 0.05), with reduced growth at both lower and higher concentrations. These results indicate that in situ feeding and growth rates are lower than laboratory rates, and that early-stage predation by ephyrae is modulated by prey availability and environmental history. Such findings provide essential baseline data for predicting the bloom dynamics and ecological impacts of *Aurelia* in temperate coastal environments.

## Simple Summary

Jellyfish often form large groups, called blooms, which can affect the health of coastal waters and the animals living there. One stage in the jellyfish life cycle, called the ephyra, is very small and hard to study in the wild, but is important because it can shape how large a bloom will become. In this study, we collected young jellyfish from two coastal bays in Korea and measured how much food they ate and how fast they grew in their natural environment. We found that jellyfish which started life in the wild ate more and grew more than those raised in the lab, and that jellyfish grew best when food was available at just the right level: not too much or too little. These findings show that the success of jellyfish blooms depends on both natural food conditions and the jellyfish’s own experience in their environment. Understanding how young jellyfish feed and grow in nature will help scientists and managers predict and possibly control jellyfish outbreaks, which can affect fishing, tourism, and marine life.

## 1. Introduction

Recent years have witnessed frequent and massive blooms of jellyfish, particularly *Aurelia coerulea*, in many coastal regions worldwide, raising considerable ecological and economic concerns due to their impacts on marine fisheries, aquaculture, and broader marine ecosystems [1,2,3]. Such blooms profoundly affect zooplankton and ichthyoplankton populations by competing for food resources with fish, and by directly preying on fish eggs and larvae, leading to negative consequences for fisheries [4,5,6,7]. Frequent occurrences of jellyfish blooms have been reported in coastal and estuarine waters of Asia, Europe, and North America, and are often attributed to climate change and anthropogenic environmental changes such as coastal development [8,9,10,11].

In the life cycle of scyphozoan jellyfish, polyp populations constitute a persistent benthic stage, reproducing asexually and releasing ephyrae through strobilation [2,3]. The abundance and spatial distribution of polyps, which are often enhanced by artificial substrates such as oyster farms, directly influence the frequency and magnitude of blooms [1,3]. Following this, the ephyra stage represents a critical recruitment bottleneck that largely determines the success of medusa development and, ultimately, the scale of population outbreaks [12,13,14,15,16]. Ephyrae primarily feed on small zooplankton such as copepod nauplii, bivalve veligers, and rotifers, and previous studies have shown that both their feeding rates and growth rates are highly sensitive to environmental variables, including prey type and availability, water temperature, salinity, dissolved oxygen, and prey concentration [6,17,18,19].

The growth and survival of ephyrae are particularly dependent on prey availability, a relationship that is especially pronounced during the early developmental stages [3,12,19]. Ephyrae are generally small, with a diameter less than 10 mm, and at first they either lack tentacles or possess only rudimentary ones [17,20]. Despite their ecological significance, the ephyra stage of *Aurelia* spp. remains relatively understudied, and quantitative information on their abundance, feeding rates, prey capture efficiency, and population-level predation impact is limited [6,21,22]. Notably, ephyrae released by strobilation tend to appear in winter to early spring, when plankton biomass is at its lowest, making the success of feeding and survival during this period a crucial determinant of population dynamics [13,19,23]. Most prior research has evaluated the feeding and growth characteristics, prey selectivity, and responses to environmental variables such as temperature, salinity, and prey concentration of jellyfish ephyrae primarily under laboratory conditions [12,18,19,24,25]. However, in the field, factors including prey community composition, plankton biomass, temperature, and microbial assemblages vary across space and time, leading to discrepancies between laboratory results and natural responses [22,26]. Moreover, quantitative data on the actual feeding rates of jellyfish in situ are indispensable for evaluating how recurring jellyfish blooms influence plankton communities and the dynamics of marine ecosystems [6,21,22,26]. Nevertheless, such field-based quantitative data from bloom-prone regions remain scarce due to the complexity of in situ experiments and the diverse range of environmental conditions.

Geoje Bay and Jaran Bay, located on the southern coast of Korea, is a representative region where mass occurrences of *A. coerulea* have been documented annually [3,27]. In these bays, *A. coerulea* medusae typically reach peak abundance from June to October, after which populations gradually decline [28]. The high abundance of zooplankton in these bays, particularly during spring and early summer, is thought to provide the nutritional foundation for the rapid growth and recruitment success of *A. coerulea* populations during bloom periods [3]. Because the early developmental ephyra stage exerts a decisive influence on the successful recruitment of adult medusae, elucidating the grazing behavior, prey selectivity, and growth characteristics of ephyrae under natural conditions is crucial for predicting bloom magnitude and assessing ecological impacts. In this study, we conducted in situ chamber experiments to quantitatively evaluate the feeding and growth dynamics of *A. coerulea* ephyrae within natural plankton communities, the patterns of prey utilization during early development, and the resulting changes in plankton community structure. These results provide a foundation for advancing our understanding of the ecological role of ephyrae and the mechanisms governing population recruitment in jellyfish bloom hotspots.

## 2. Materials and Methods

### 2.1. Collection and Rearing of Polyps and Ephyrae

**Figure 1:**
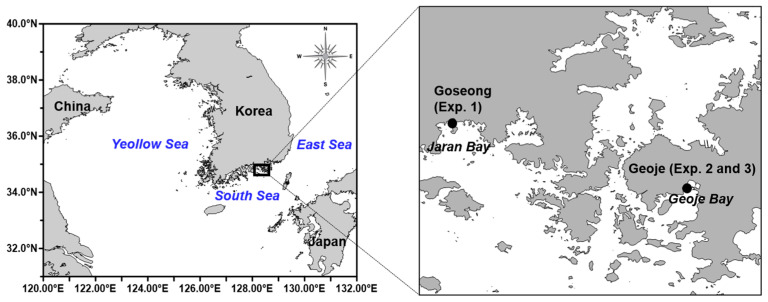
Location of the experimental sites in Jaran Bay (Exp. 1) and Geoje Bay (Exp. 2 and 3) on the southern coast of Korea.

Geoje Bay and Jaran Bay are semi-enclosed bays located on the eastern South Sea coast of Korea (Figure 1), supporting a high diversity of marine organisms. Both bays are designated as fisheries resource conservation areas and are sites of intensive aquaculture, including oysters, fish, and sea squirts. Suspended longline oyster aquaculture is especially prominent in these bays; Geoje Bay has been certified as a shellfish growing area by the U.S. Food and Drug Administration (FDA) [29]. Jaran Bay similarly supports extensive shellfish mariculture and has been the focus of long-term studies on primary productivity and environmental factors related to shellfish farming [30]. A field survey conducted on 3 April 2025, revealed that ephyrae were absent in Geoje Bay at the time of sampling. Consequently, for the first in situ experiment, field ephyrae were collected from the adjacent Jaran Bay, located in close proximity to Geoje Bay and characterized by similar hydrographic features. For all subsequent field and laboratory experiments, either ephyrae collected directly from Geoje Bay or those reared in the laboratory were used, as described in the following sections. Polyps of *A. coerulea* were obtained from underwater collections at oyster farms in Geoje Bay, Korea (collection date: April 2023). The collected polyps were maintained at the NIFS in filtered seawater (20 μm) at 10 °C and 32 PSU under a 12 h light–12 h dark photoperiod (light intensity: 50 μmol photons m^−2^ s^−1^). Fresh *Artemia* nauplii were provided as food once per day at a rate of ~100 nauplii per polyp. All cultured polyps were confirmed as *A. coerulea* via genetic analysis (mtCOI, 16S rRNA partial sequences) [3].

Only newly released ephyrae from these polyps were used in the experiments. Prior to experimentation, ephyrae were acclimated for 24 h in situ seawater. After acclimation, only individuals with empty gastrovascular cavities (i.e., no visible food remnants) were selected for subsequent trials.

### 2.2. Experimental Setup

Experiments were conducted using 0.5 L cylindrical acrylic chambers (internal diameter: 12 cm; height: 25 cm) deployed on buoy platforms in Geoje Bay (34 °8′ N, 128 °57′ E). Chambers were filled with freshly collected in situ seawater (Trial 1: 12.1 °C, 33.3 PSU; Trial 2: 14.1 °C, 33.1 PSU; Trial 3: 15.1 °C, 33.3 PSU). At the time of sampling, chlorophyll *a* concentrations were 2.6 μg L^−1^ in Jaran Bay and 2.1–2.3 μg L^−1^ in Geoje Bay, which was indicative of moderate phytoplankton biomass in both areas. Surface seawater samples (0–1 m depth) were collected using an 8 L PVC Niskin bottle. For Chl-*a* analysis, 0.5 L of seawater was filtered through a 0.45 μm membrane filter (47 mm, ADVANTEC, Japan) using a filter holder. The filters were immediately stored at −80 °C and transported to the laboratory. Chl-*a* concentrations were extracted by immersing the filters in 90% acetone for 24 h at 4 °C in the dark. After extraction, the solution was filtered with a 0.45 μm PTFE syringe filter (ADVANTEC, Tokyo, Japan) to remove any particulates. Chl-*a* concentrations were determined using a calibrated fluorometer (10-AU, Turner Designs, San Jose, CA, USA) with standard Chl-*a* (Sigma, St. Louis, Mo, USA) for instrument calibration. For each trial, 5–10 ephyrae were introduced per chamber. For the first experiment, ephyrae collected directly from the field were assigned to two size categories (small: 1.7–4.0 mm; large: 4.8–7.5 mm), according to their central disc diameter (CDD) or, where applicable, total disc diameter (TDD). The boundaries for small and large size groups were defined based on the size distributions documented in earlier studies [18,19,24], as well as the size-frequency characteristics of the field population in the present work. For each experimental condition, three chambers were established in parallel as biological replicates, each set up with identical initial prey concentrations, chamber volumes, and ephyra densities. Accordingly, all subsequent analyses were conducted using *n* = 3 per treatment, except where noted. In the first trial (2 April), small ephyrae were sufficiently abundant to permit four replicate chambers, whereas the large ephyra group comprised only two chambers due to limited specimen availability. Initial size distributions were determined by measuring at least 20 individuals prior to each experiment. Each ephyra was photographed using a stereomicroscope (Nikon SMZ18, Nikon, Tokyo, Japan) equipped with a digital camera (Nikon DS-Ri2, Nikon, Tokyo, Japan). Morphometrics (vertical and horizontal axes, μm) were precisely determined using NIS-Elements software (ver. 3.0, Nikon Instruments Inc., Melville, NY, USA).

**Figure 2:**
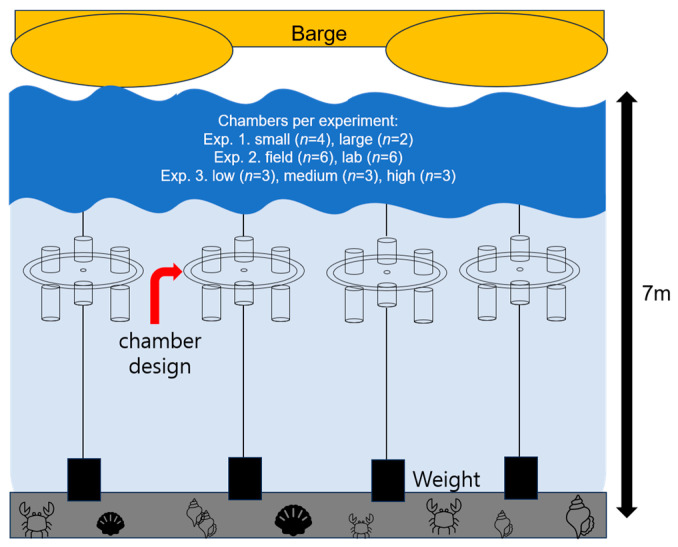
Experimental setup of in situ chambers.

All experimental chambers containing ephyrae and prey zooplankton were incubated in situ for 24 h (Figure 2). To prevent prey sedimentation and ensure homogeneous prey distribution within chambers, a 60 μm mesh was installed along the inner walls of each chamber, facilitating gentle water circulation. At the beginning and end of the incubation, all chamber content was carefully retrieved, and the remaining nauplii, copepodites, adults, and ephyrae were enumerated under a stereomicroscope. Ephyra size, survival, and any morphological damage or abnormalities were documented immediately after experiments using digital images captured via a stereomicroscope (Nikon SMZ18, Nikon DS-Ri2) and analyzed using NIS-Elements software.

### 2.3. Prey Organisms

Copepods (nauplii (body length: 79.2–217.5 μm), copepodites (body length: 314.5–611.7 μm), and adults (body length: 820.8–970.6 μm)) used as prey were collected from the surface to bottom layers in Jaran Bay and Geoje Bay using a 60 μm mesh conical plankton net (net mouth diameter: 30 cm). Sampling was conducted via oblique tows at a speed of 2 knots for 2 min on three occasions (2 April, 14 April, and 23 April 2025). Immediately after collection, samples were transferred to 1000 mL plastic containers and transported under refrigeration. After concentration in a 50 mL conical tube, the samples were split into two aliquots, each thoroughly mixed, and subsequently pooled to prepare a homogenous suspension for initial counting and distribution to experimental chambers.

To determine initial prey concentrations, 1 mL subsamples were taken from the pooled suspension and counted under a stereomicroscope (10 replicates per sample). Initial abundances of nauplii, copepodites, and adults, as well as total carbon concentrations, were calculated based on these counts. The prey suspension was then distributed into each experimental chamber to achieve target initial prey carbon concentrations ranging from 200 to 800 μg C L^−1^. Most previous studies adjusted prey biomass at the chamber or beaker level, whereas in this study, the initial prey carbon input was standardized per individual ephyra. By controlling the available prey biomass for each ephyra, we were able to directly estimate individual feeding potential and minimize confounding effects arising from differences in animal density. The per capita prey input was determined based on zooplankton densities reported in previous studies [18,19]. The carbon content of each zooplankton group was calculated using established conversion factors (μg C ind.^−1^). Lengths of at least 30 individuals per group were measured to determine the mean carbon content.

For copepodites and adults, individual carbon content (C, μg C ind^−1^) was estimated from body length (L, μm) using the equations provided by Uye [31,32]:

copepodites: C = L^3.07^/10^8.37^

adults: C = L^3.08^/10^8.51^

nauplii: C = 1.51 × 10^−5^ × L^2.94^

where L is the total length (μm) and C is the individual carbon content (μg C ind.^−1^).

### 2.4. Grazing and Growth Parameters

Grazing efficiency (%) was calculated as follows [19]:

Grazing efficiency (%) = (Grazing C (μg)/Expected Intake C (μg)) × 100

The final prey carbon concentration (Ct, μg C L^−1^) was calculated as [19]:

Ct = Total Remain C (μg)/Chamber Volume (L)

**Figure 3:**
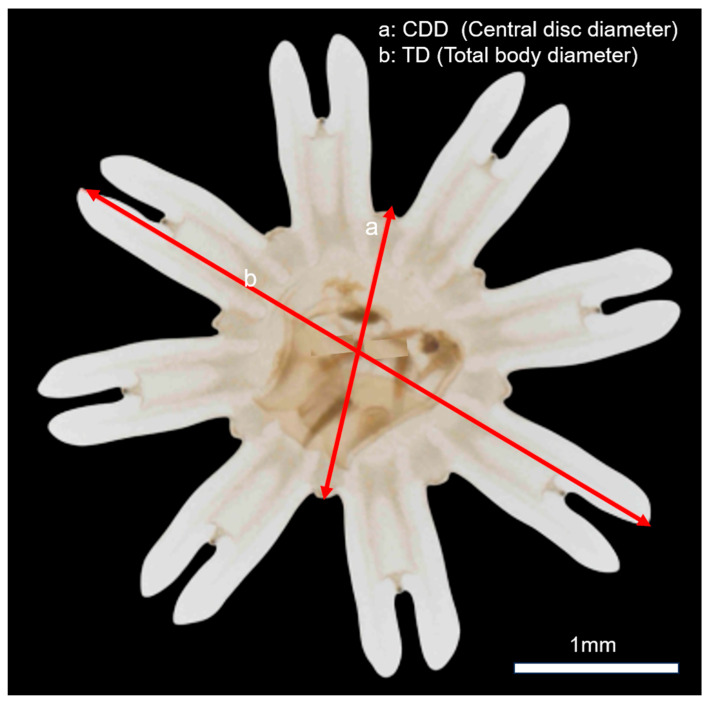
Measurement scheme for *Aurelia coerulea* ephyra. (a): central disc diameter (CDD); (b): total body diameter (TD).

Specific Growth Rate (SGR, day^−1^) was determined as [24]:

SGR = (ln(Size_after/Size_before))/T

where size_before and size_after are the average central disc diameters (CDDs, mm) measured before and after the 24 h incubation (Figure 3), and T is the incubation time (days).

The clearance rate (CR, mL ind^−1^ h^−1^) was calculated according to Frost [33], and Kamiyama [19]:

CR = ((lnC_0_ − lnCₜ)/t) × (V/n)

where C_0_ and C_t_ are the initial and final prey carbon concentrations (μg C mL^−1^), t is the incubation duration (h), V is the chamber volume (mL), and n is the number of ephyrae per chamber.

### 2.5. Statistical Analysis

Nonparametric statistical methods were employed to evaluate differences in specific growth rate (SGR), grazing efficiency, clearance rate, and grazing carbon per ephyra among experimental groups and food concentration treatments. All analyses were conducted in R version 4.4.2 [34] using the ‘rstatix’, ‘ggpubr’, ‘tidyverse’, ‘rcompanion’, and ‘effsize’ packages.

In Exp. 1, differences in SGR, grazing efficiency, clearance rate, and grazing carbon per ephyra between field-collected ephyrae categorized as small (TD: 1.7–4.0 mm) and large (TD: 4.8–7.5 mm) were assessed using the Wilcoxon rank sum test (nonparametric Mann–Whitney U test), as the sample sizes were small (*n* = 4 and *n* = 2, respectively) and assumptions of normality and homoscedasticity were violated. Cliff’s delta was calculated as a measure of effect size.

In Exp. 2, differences in SGR, grazing efficiency, clearance rate, and grazing carbon per ephyra between field-collected and laboratory-reared ephyrae (*n* = 6 per group) were also evaluated using the Wilcoxon rank sum test and Cliff’s delta. All results are presented as mean ± standard deviation (SD).

In Exp. 3, the effects of food concentration (56 ± 3.5, 122 ± 11.4, and 242 ± 0 μg C L^−1^, corresponding to low, medium, and high treatments, respectively) on SGR, grazing efficiency, clearance rate, and grazing carbon per ephyra in laboratory-reared ephyrae were analyzed using the Kruskal–Wallis test. Epsilon squared (ε^2^) was calculated as an index of effect size. When a significant difference (*p* < 0.05) was detected, Dunn’s post hoc test was applied for multiple pairwise comparisons between groups. Statistical significance (*p*-values) is indicated in all tables and figures, and all interpretations fully consider the limitations imposed by small sample sizes.

## 3. Results

### 3.1. Exp. 1: Size-Specific Feeding and Growth

**Figure 4:**
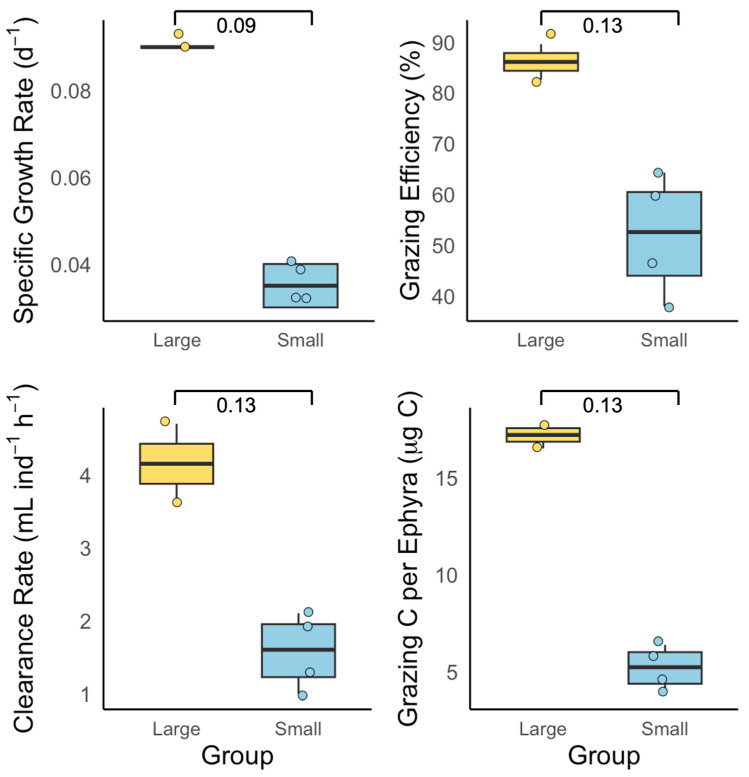
Feeding responses of field-collected *Aurelia coerulea* ephyrae categorized by size: large (yellow) and small (blue).

Field-collected ephyrae were categorized as small (1.7–4.0 mm, *n* = 4) or large (4.8–7.5 mm, *n* = 2). The mean SGR was 0.036 ± 0.001 d^−1^ for small and 0.088 ± 0.001 d^−1^ for large ephyrae, with borderline significance (Wilcoxon rank sum test, *p* = 0.09; Figure 4). The grazing efficiency was 51.9 ± 12.0% for small and 85.8 ± 5.0% for large ephyrae. The clearance rate averaged at 1.6 ± 0.5 mL ind^−1^ h^−1^ (small) and 4.2 ± 0.8 mL ind^−1^ h^−1^ (large). The mean grazing carbon per ephyra was 5.2 ± 1.1 μg C (small) and 17.2 ± 1.0 μg C (large).

### 3.2. Exp. 2: Field-Collected Versus Laboratory-Reared Ephyrae

**Figure 5:**
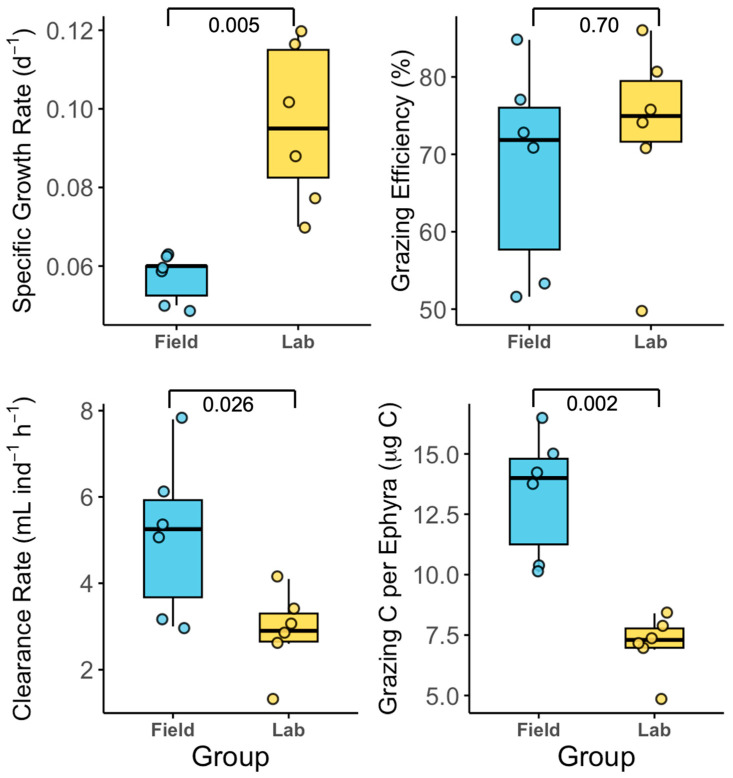
Feeding responses of *Aurelia coerulea* ephyrae derived from field collections (blue) or laboratory rearing (yellow).

Field-collected and laboratory-reared ephyrae exhibited mean SGR of 0.056 ± 0.004 d^−1^ and 0.097 ± 0.019 d^−1^, respectively, with significant difference between groups (Wilcoxon rank sum test, *p* < 0.01; Figure 5). The grazing efficiencies were 68.4 ± 12.3% (field) and 72.7 ± 12.9% (lab), with no significant difference (*p* > 0.05). Clearance rates were higher in field-collected ephyrae (5.1 ± 1.7 mL ind^−1^ h^−1^) than in laboratory-reared ephyrae (2.9 ± 0.9 mL ind^−1^ h^−1^; *p* <0.05). The amount of grazing carbon per ephyra was 13.9 ± 2.7 μg C (field) and 7.1 ± 1.4 μg C (lab) (*p* < 0.01).

### 3.3. Exp. 3: Effects of Food Concentration on Laboratory Ephyrae

**Figure 6:**
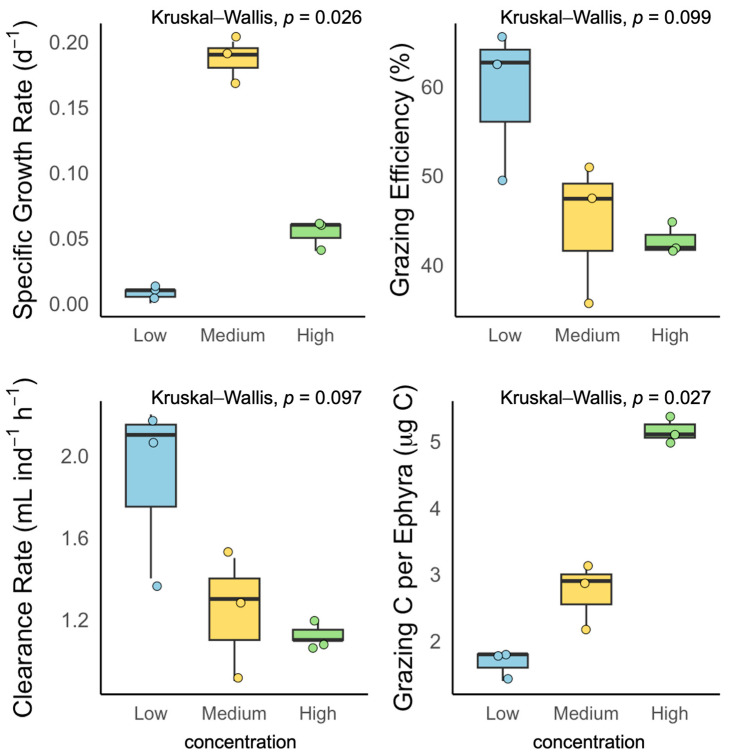
Feeding responses of laboratory-reared *Aurelia coerulea* ephyrae incubated in situ at three food concentrations: low (blue), medium (yellow), and high (green).

Laboratory ephyrae were incubated at low (56 ± 3.5 μg C L^−1^), medium (122 ± 11.4 μg C L^−1^), or high (242 ± 0 μg C L^−1^) food concentrations (*n* = 3 each). The SGR differed significantly among treatments (Kruskal–Wallis test, *p* < 0.05; Figure 6), with mean values of 0.0077 ± 0.004 d^−1^ (low), 0.189 ± 0.013 d^−1^ (medium), and 0.053 ± 0.011 d^−1^ (high). The grazing efficiency was 59.2 ± 8.6% (low), 44.6 ± 7.9% (medium), and 42.7 ± 1.8% (high) (Kruskal–Wallis test, *p* > 0.05, ε^2^ = 0.42). The clearance rate was highest at a low food concentration (1.9 ± 0.4 mL ind^−1^ h^−1^), declining at medium (1.3 ± 0.3 mL ind^−1^ h^−1^) and high (1.2 ± 0.1 mL ind^−1^ h^−1^) concentrations (*p* > 0.05, ε^2^ = 0.40). Grazing carbon per ephyra increased with food concentration: 1.7 ± 0.2 μg C (low), 2.7 ± 0.5 μg C (medium), and 5.2 ± 0.2 μg C (high) (Kruskal–Wallis test, *p* < 0.01, ε^2^ = 0.83).

## 4. Discussion

### 4.1. Comparison of Growth and Feeding Efficiency: Size-Dependent

In this study, field-measured growth rates, per-individual clearance rates, and grazing carbon were substantially lower than those reported in laboratory experiments. For example, Riisgård & Madsen [35] observed clearance rates of 38–59 mL h^−1^ for 5 mm *Aurelia aurita* ephyrae fed *Artemia* nauplii in the lab, but rates declined sharply to 0.8–45.6 mL ind^−1^ h^−1^ when copepod nauplii or adults, which exhibit strong escape behavior, were used as prey. Thus, laboratory feeding rates markedly differ depending on prey type, whereas in nature, the diversity of prey and their escape behaviors, as well as environmental fluctuations, may restrict feeding and growth [17,18]. In this study, the field experiments used natural plankton communities (copepod nauplii, copepodites, and adults) as prey instead of artificial foods like *Artemia*, resulting in lower clearance rates and grazing carbon than laboratory maxima and high variability among individuals. The dominant prey in these experiments were copepod nauplii, copepodites, and adults, all of which are less easily captured than *Artemia*. Sullivan et al. [17] also showed that ephyrae captured actively swimming prey such as *Artemia* or rotifers more effectively than copepod nauplii, which tend to escape or feign death [17]. Previous studies have also indicated that container effects, limited mixing, and differences in prey composition and concentration between laboratory and field conditions may affect actual feeding rates [16,19,24,26,35]. The in situ chamber experiments in this study aimed to reflect real plankton prey and mixing conditions, but the lower feeding and growth rates observed relative to laboratory studies indicate that prey escape, patchiness, low prey concentrations, and various environmental stressors (water temperature, salinity, and ocean current) all likely contributed to field outcomes [18].

Larger ephyrae generally exhibited higher SGR, clearance rates, and grazing carbon than smaller individuals, demonstrating the ecological advantages of increased size. Low growth rates and grazing efficiency were consistently observed in small individuals. While the numerical differences between size classes were often substantial, statistical significance was detected only in Exp. 2 (Figure 5), where the sample size was larger; in Exp. 1 (Figure 4), limited replication likely precluded the detection of significant effects. These results suggest that increased prey capture efficiency, energetic use, and the ability to handle escaping prey in larger ephyrae can produce real differences in growth [12,19,24,27]. The ecological consequences of size-based differences are likely mediated by multiple factors, including stage duration, mortality, cohort progression, and temporal variability in prey fields. To fully resolve the functional significance of size structure in natural populations, future studies should address limitations in replication and sampling scale.

### 4.2. Comparison of Growth and Feeding Efficiency: Field-Collected and Laboratory-Reared Ephyrae

Field-collected ephyrae consistently showed higher clearance rates (*p* < 0.05) and individual grazing carbon (*p* < 0.01) than laboratory-reared ephyrae under the same in situ conditions. However, laboratory-reared ephyrae exhibited a significantly higher SGR (*p* < 0.01), while grazing efficiency did not differ significantly (*p* > 0.05). This indicates that both experimental conditions and previous exposure to prey diversity, environmental variability, and stress can influence the functional responses of ephyrae [12,18].

Field-collected ephyrae may develop greater behavioral and physiological flexibility by being exposed to diverse *prey* assemblages, variable temperature and salinity, and prey escape behaviors. In contrast, laboratory-reared individuals, which are exposed only to uniform prey (mainly *Artemia*) and stable environments, exhibit a narrower adaptive range and reduced foraging flexibility. These differences in environmental history may lead to over- or underestimation of ecological function in laboratory settings. The high clearance rates observed in field ephyrae are likely due to experience with actively swimming, escape-prone prey such as copepod nauplii and copepodites, which are common in natural environments. Laboratory-reared ephyrae may lack such adaptive experience [18]. Environmental and trophic variability in the field underpins differences in nutritional plasticity and functional responses of jellyfish between laboratory and natural environments [36]. For example, the clearance rate and predation impact of *A. coerulea* measured in the laboratory can differ from field values depending on prey composition and environmental factors [18]. Other studies have also shown that the physiological and behavioral traits of laboratory-reared individuals may not sufficiently reflect the functional traits needed for population maintenance and successful settlement in natural environments [24,37]. Therefore, results based on laboratory-reared ephyrae should be interpreted with caution when applied to natural population dynamics, plankton community effects, or recruitment success [24,37].

**Figure 7:**
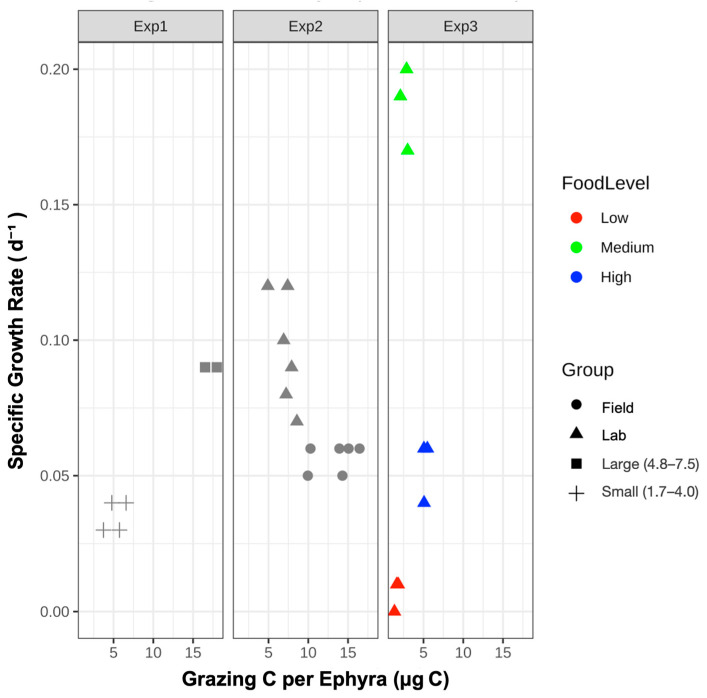
Relationship between specific growth rate (SGR) and grazing carbon per ephyra across three in situ experiments (Exp1: 12.1 °C, Exp2: 14.1 °C, Exp3: 15.1 °C). Symbols indicate group (field, lab, large, and small) and food level (low, medium, and high).

Temperature is recognized as a major factor influencing the growth and metabolic activity of jellyfish ephyrae [24,25]. In this study, the three experiments were conducted at progressively higher temperatures (12.1, 14.1, and 15.1 °C), and SGR and grazing carbon showed variable responses across trials (Figure 7). The highest SGR was observed in the experiment at the highest temperature, while grazing per ephyra appeared to be more strongly influenced by individual size. However, interpretation is complicated by simultaneous differences in ephyra size, origin, and food availability among experiments. Nevertheless, the observed trends are generally consistent with previous reports of temperature-dependent growth in scyphozoans, but our results indicate that ephyra growth and feeding are shaped by interactions between environmental temperature, food supply, and body size.

### 4.3. Comparison of Growth and Feeding Efficiency: Prey Concentration

Experiments manipulating prey concentration revealed that feeding and growth parameters, including SGR and grazing carbon, displayed nonlinear responses. SGR peaked at intermediate prey concentrations, whereas both low and high prey levels led to pronounced reductions in growth and feeding efficiency. In contrast, grazing carbon per ephyra increased steadily with food supply, while grazing efficiency and clearance rate tended to plateau or decline at higher prey densities. This pattern reflects the operation of multiple ecological mechanisms, such as optimal foraging, feeding saturation, and prey interference [12,18,19].

At low concentrations, feeding and growth are constrained by low encounter rates with prey, while high concentrations induce decreased feeding efficiency due to satiation, prey patch formation, and interference among prey organisms [12,37]. Notably, variance in individual growth rates was maximal at intermediate concentrations, suggesting that beyond mean prey availability, fine-scale features of the prey environment may influence population-level outcomes. In addition to prey concentration, the composition and size structure of available prey were found to be decisive factors influencing feeding and growth. Ephyrae primarily consumed smaller prey such as nauplii, but larger and more developed individuals showed increased consumption of copepodites and adults.

Ephyrae exhibit strong prey selectivity [17,19], and feeding and growth are maximized when planktonic ciliates with low escape ability dominate the community, typically during the initial post-release phase or when small zooplankton are abundant. Such seasonal shifts in community composition can directly influence the growth and recruitment window for jellyfish blooms [6,19,38,39]. However, it is well established that ciliates and other microplankton are essential for newly released ephyrae, which have limited feeding and growth capabilities [17,19]. In Exp. 1, nauplii comprised most of the prey consumed by both small (mean 88.5 ± 12.6%) and large (90.0 ± 4.2%) ephyrae. However, adults accounted for only a mean of 27.5 ± 34.0% of prey in the small group, but a mean of 60 ± 28.3% in the large group, indicating that larger ephyrae are able to capture and consume a greater proportion of adult prey as they develop. Overall, these findings highlight that the actual diversity and spatiotemporal variability of the prey field fundamentally modulate the physiological performance of ephyrae, and must be incorporated into interpretations of their feeding traits [6,27]. Under optimal conditions, ephyrae can influence zooplankton community structure directly [3,18], potentially contributing to ecosystem resilience and shifts in trophic networks.

Based on observed field densities (0.5–5.3 inds. m^−3^ in Geoje Bay; 164 inds. m^−3^ in Jaran Bay) and measured clearance rates, the estimated daily removal of zooplankton by *A. coerulea* ephyrae ranged from 0.004% to 0.10% in Geoje Bay and from 0.39% to 1.85% in Jaran Bay. These values are much lower than the population clearance rates reported for jellyfish in other studies (Table S1 in the Supplementary). Therefore, the grazing impact of ephyrae is highly context-dependent, varying considerably with prey availability, prey community structure, the timing and magnitude of jellyfish recruitment, and the size structure of the ephyrae population.

## 5. Conclusions

This study identified the feeding and growth characteristics of *A. coerulea* ephyrae under in situ conditions. Larger ephyrae exhibited significantly higher feeding and growth efficiencies than smaller individuals (Figure 4), and field-collected ephyrae showed notably greater SGR, clearance rates, and carbon ingestion compared to laboratory-reared individuals (Figure 5). SGR peaked at intermediate levels, whereas grazing carbon increased with prey supply, and clearance rate and efficiency either leveled off or declined at higher densities (Figure 6). These results demonstrate that the feeding and growth of *A. coerulea* ephyrae are shaped by both traits (size and origin) and environmental conditions (prey availability), and that different growth and feeding parameters respond distinctly to food supply. Consequently, data obtained from laboratory-reared ephyrae alone may not adequately represent the predation impact or bloom dynamics of natural populations.

## Data Availability

The raw data supporting the conclusions of this article will be made available by the authors on request.

## Supplementary Materials

The following supporting information can be downloaded at: https://www.mdpi.com/article/10.3390/biology14060687/s1, Table S1: Summary of Experimental Conditions and Clearance Rates (mL ind^−1^ h^−1^) [18,21,35,37,40,41].
