# Supplementary material for: Feeding and Growth in the Ephyra Stage of Aurelia coerulea: An In Situ Study
Source: Biology (Basel). 2025 Jun 12;14(6):687. doi: 10.3390/biology14060687 (PMC12190127; doi:10.3390/biology14060687)
Supplement: Supplementary file 1 [file biology-14-00687-s001.zip › biology-3694837-supplementary.pdf]

Table S1. Summary of Experimental Conditions and Clearance Rates (mL ind^{-1} h^{-1})

Species	Prey Type	Temp. (°C)	Clearance Rate (mL ind^{-1} h^{-1})	Reference
Aurelia coerulea **(field, small)**	copepods nauplii+coepodites+adults	12.1	1.0–2.14	**This study**
Aurelia coerulea **(field, large)**	copepods nauplii+coepodites+adults	12.1	3.64–4.71	**This study**
Aurelia coerulea **(field)**	copepods nauplii+coepodites+adults	14.1	3.03–7.84	**This study**
Aurelia coerulea **(lab)**	copepods nauplii+coepodites+adults	14.1	1.44–4.1	**This study**
Aurelia coerulea **(lab, low)**	copepods nauplii+coepodites+adults	15.1	1.42–2.22	**This study**
Aurelia coerulea **(lab, med)**	copepods nauplii+coepodites+adults	15.1	0.92–1.48	**This study**
Aurelia coerulea **(lab, high)**	copepods nauplii+coepodites+adults	15.1	1.11–1.24	**This study**
Aurelia coerulea	copepods	18.3–18.8	8–488	**Wang et al. 2020 [18]**
Aurelia coerulea	hydromedusae	18.8–18.9	61–1580	**Wang et al. 2020 [18]**
Aurelia aurita	*Brachionus plicatilis*	14–16	7.8–10.8	**Olesen et al. 1994 [21]**
Aurelia aurita	*Acarita tonsa*	10–20	258–272	**Olesen et al. 1994 [21]**
Aurelia aurita	copepods	15	1–54	**Riisgård and Madsen, 2011 [35]**
Aurelia aurita	*Artemia*	15	377–3800	**Riisgård and Madsen, 2011 [35]**
Aurelia aurita	copepod nauplii	12.4	3.8–45.6	**Riisgård and Madsen, 2011 [35]**
Aurelia aurita	*Artemia*	12.4	3.8–85.2	**Riisgård and Madsen, 2011 [35]**

Aurelia aurita	Rotifer	12.4	10.6–33.6	**Riisgård and Madsen, 2011 [35]**
Aurelia aurita	copepods (Adults)	12.4	0.8	**Riisgård and Madsen, 2011 [35]**
Chyrsaora quinquercirrha	*Synchaeta* sp.	23–24	1.3	**Olesen et al. 1996 [37]**
Chyrsaora quinquercirrha	copepod nauplii	23–24	0.3	**Olesen et al. 1996 [37]**
Chyrsaora quinquercirrha	Tintinnids	23–24	0.25	**Olesen et al. 1996 [37]**
Aurelia aurita	>50 um microplankton	15	50–558	**Stoecker et al. 1987 [40]**
Pelagia noctiluca	Tuna eggs	23	4140	**Gordoa et al. 2013 [41]**
